# Intragenic recombination between two non-functional *semi-dwarf 1* alleles produced a functional *SD1* allele in a tall recombinant inbred line in rice

**DOI:** 10.1371/journal.pone.0190116

**Published:** 2017-12-27

**Authors:** Bi Wu, Wei Hu, Mohammed Ayaad, Hongbo Liu, Yongzhong Xing

**Affiliations:** 1 National Key Laboratory of Crop Genetic Improvement, Huazhong Agricultural University, Wuhan, China; 2 Nuclear Research Center, Egyptian Atomic Energy Authority, Cairo, Egypt; Institute of Genetics and Developmental Biology Chinese Academy of Sciences, CHINA

## Abstract

Intragenic recombination is one of the most important sources of genetic variability. In our previous study, RI92 a tall line (160 cm of plant height) was observed in the cross progeny between two semi-dwarf *indica* cultivars Zhenshan 97 and Minghui 63. Genome-wide genotyping and sequencing indicated that the genome constitution of RI92 was completely from both parents. Bulk segregant analysis in a BC_3_F_2_ population revealed that “green revolution gene” *semi-dwarf 1* (*sd1*) was most likely the gene controlling the tall plant height in RI92. Sequencing analysis of *SD1* revealed that an intragenic recombination occurred between two parental non-functional *sd1* alleles and generated a functional *SD1* in RI92. Four-fold high recombination rate in *SD1* located bins to the genome-wide average was observed in two RIL populations, indicating recombination hotspot in the *SD1* region. Intragenic recombination creates new alleles in the progeny distinct from parental alleles and diversifies natural variation.

## Introduction

Recombination and mutation are the motivation of evolution. Recombination is critical for repairing DNA lesions and for chromosomal pairing and exchange during meiosis [1]. The well-studied yeast meiotic recombination revealed that non-uniformity of recombination is observed when the frame of reference is an entire chromosome, a multi-gene region, a pair of genes, or a small region upstream of a gene [2]. The ratio of genetic distance, measured by meiotic recombination frequencies, to physical distance, measured by molecular markers, is used for evaluating recombination rate and varies within different species and genome regions. The genome regions with higher recombination rate than the genome average is called recombination hotspots, which enhance the resolution of gene mapping and help introduce the excellent alleles into breeding lines by broken linkage. Intragenic recombination, the recombination among different varying sites within the same gene locus, can create novel alleles with more genetic variability, and benefit to evolution which selection could act [3]. To identify intragenic recombination, restriction fragment length polymorphism (RFLP) markers within target genes were used to distinct two parental alleles in traditional genetics [4]. The intragenic recombination causes a distinct fragment pattern from both parents. Intragenic recombination hotspots have been observed at a number of gene loci in the recombination hotspots in plants, such as the *wx* locus in rice *(Oryza sativa*) and maize (*Zea mays*); the maize *A1* locus and the bronze locus [4–7]. The recombination rate at *A1* locus was from 12.5 kb/cM to 25 kb/cM, which is two orders of magnitude higher than the average rate of whole maize genome [5]. However, not all the intragenic recombination was occurred in the recombination hotspots, only by chance. For example, the intragenic recombination rate at *csr1* locus in *Arabidopsis* is almost the same as the genome average [8]. To easily discover the intragenic recombination, two individuals carried non-functional alleles of targeted genes; whose intragenic recombination causes a functional allele and obtained a distinct phenotype change, are priority used to develop a population for estimation of intragenic recombination rate [9, 10]. The functional alleles/plants in the progeny are resulted from intragenic recombination, and the ratio of functional allele plants to mutation allele plants was deemed as intragenic recombination rate. For example, the *wx* gene encodes a granule-bound starch synthase required for the synthesis of amylose in both the endosperm and pollen of maize and rice *Wx* pollen grains stain deep blue in I_2_-KI solution, whereas *wx* pollen grains stain light brown. The large sample of pollen and the easily phenotype distinguishing with I_2_-KI staining make *wx* frequently used for estimating intragenic recombination rate [4]. The progeny of the hybrid between two *wx* mutants each from different mutation events produces *Wx* pollen, which is the result of intragenic recombination of *wx* alleles. The recombination frequency within the *wx* gene was 10 times higher than the genome average [6]. With the advent of next generation sequencing technique, high-density bin maps were constructed with RILs populations in maize and rice [11–13]. Intergenic and intragenic recombination could be detected on the basis of allele sequence, and significant associations between intragenic recombination events and agronomic traits were observed [13].

Several studies support the hypothesis that recombination occurs primarily in genes: (1) comparing to gene-poor chromosomal regions, the gene-rich regions are recombination active in wheat and barley [14, 15], (2) in Arabidopsis, maize and rice, recombination is suppressed in the regions nearby centromere [16–18]. The higher recombination frequencies in maize were predictable on the broad scale, following the distribution of gene density and CpG methylation; meanwhile, the inversions also suppress recombination in distinct regions [18]. Rice genome exhibits recombination hot spot and cold spot, where recombination rates per kb are significantly higher and lower than the genome average [19, 20].

Plant height, one of the most important traits affecting rice production, was widely researched in the recent years. There are various factors regulating plant height, but gibberellin (GA) is the most important factor. *SD1* as the green revolution gene encoded gibberellin 20-oxidase and involved in GA synthesis[21]. It is a key enzyme in gibberellin biosynthesis that catalyzes the three steps; GA53**→**GA44**→**GA19**→**GA20. Impaired GA 20-oxidase activity will accumulate the content of GA53, and the reduced amount of GA20 resulted in a decreased plant height. At least, there are seven *sd1* alleles have been used in the breeding of semi-dwarf rice varieties in China, USA and Japan [22]. Two functional nucleotide polymorphisms for the short culm of Nipponbare (*O*. *sativa* ssp. *japonica*) were identified in the coding region of *SD1*, and the dramatic nucleotide diversity reduction around *SD1* was only occurring in the japonica landraces which indicated *SD1* had been subjected to the japonica domestication [23]. These results indicated that *SD1* was not only involved in the green revolution of modern breeding but also in the early steps of rice domestication.

In our previous research, we identified an extraordinary tall line, RI92 (160 cm), in the recombinant inbred line (RIL) population during the cross between Zhenshan 97 (ZS97) and Minhui 63 (MH63). To uncover why RI92 was much taller than parents, we constructed a near-isogenic line with tall plant height using RI92 as a donor to backcross with ZS97. Map-based cloning revealed that *SD1* is the player responsible for such tall plant height in RI92. Comparative sequencing showed that intragenic recombination between two non-functional *sd1* alleles in parents resulted in a functional *SD1* in RI92.

## Materials and methods

### Plant materials and phenotypic data collection

**Fig 1:**
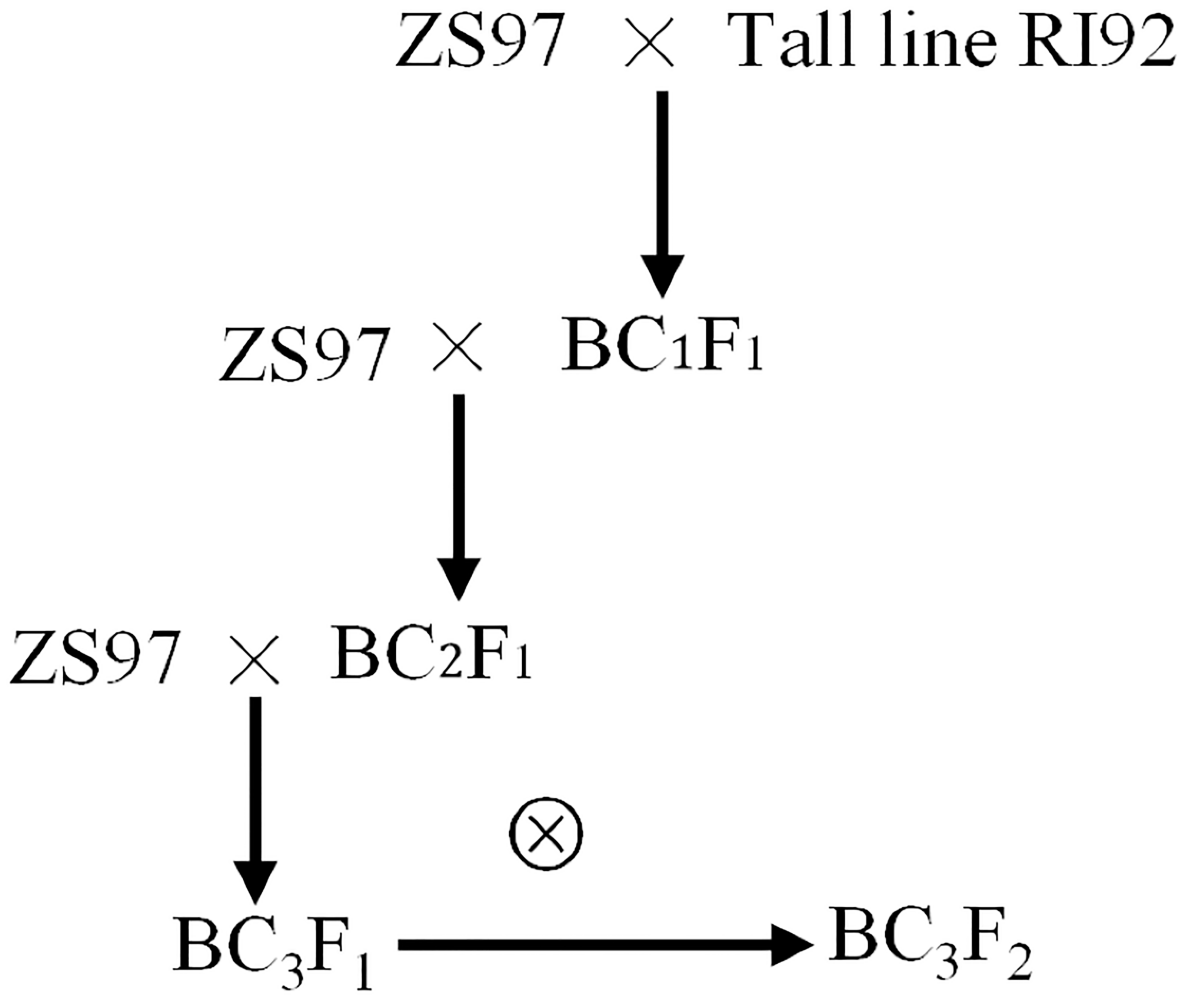
The construction of near isogenic line population used for mapping plant height.

RI92 was an extraordinary tall line of the RIL population from the cross between ZS97 and MH63 [24]. The F_1_ between RI92 and ZS97 exhibited a similar plant height to RI92. The tall plant at each backcross generation was continuously backcrossed to ZS97 two times to generate a BC_3_F_1_ plant, and then self-crossing to obtain a BC_3_F_2_ population was done (Fig 1).

The seeds of 96 BC_3_F_2_ plants were sown on 26 May 2015, then the seedlings were transplanted individually in the field at distance of 20*25 cm on 15 June 2015 in the bird net-equipped field in the experimental farm of Huazhong Agricultural University, Wuhan, China (114° 21΄ E, 30° 28΄ N). Plant height was measured in both seedling stage and maturing stage as the distance from the ground surface to the tip of the tallest leaf and the tip of the tallest panicle, respectively. At maturation stage, the whole plants individually harvested, panicle length was measured as the length from first branch node to the tip of panicle, spikelets per panicle (SPP), grains per panicle (GPP) and yield per plant (YP) were measured using automatic phenotyping machine [25]. Then harvest index was calculated as the ratio of grain weight per plant to biomass per plant.

### DNA extraction and sequencing

DNA was extracted from fresh leaves at the seedling stage using the CTAB method with minor modified [26]. *SD1* Genomic DNA including exon and intron were amplified from genomic DNA using LA *Taq* (Takara). Polymerase chain reactions (PCR) performed with the following conditions for thermal cycling: 94°C for 4 min; then 35 cycles of 94°C for 30 s, 55°C for 30 s, 72°C for 1 min per 1000-base; followed by 72°C for seven min. All primers used for PCR and sequencing listed in S1 Table. For sequencing, 5 μL PCR product digested with five U EXOI (Biolabs) and 0.13 U Shrimp Alkaline Phosphatase (Takara) together with 1×PCR buffer and incubated at 37°C for 1 hour; the reaction was stopped by maintaining the PCR product in 80°C for 20 min. Sequencing was independently performed three times in both forward and reverse primers on ABI 3730 with BigDye terminator sequencing kits (Applied Biosystems). Sequence contigs assembled by SEQUENCHER 4.1.2 (Gene Codes Corporation).

### Gibberellin acid and PBZ treatment in the seedling stage

The GA3 was first dissolved in a small volume of ethanol and diluted to 1 mM using double-distilled water (DDW). The GA3 solution was added to the rooting culture medium with a final concentration 0.2 uM, 0.4 uM, 0.8 uM and 1.6 uM. The rooting culture medium containing an equivalent amount of ethanol was used as a control. PBZ, acting by inhibiting gibberellin biosynthesis, dissolved in DDW, and the solution added to the rooting culture medium with a final concentration 0.2, 0.6 and 1.8 uM. The rooting culture with equivalent amount of DDW was used as a control.

The seeds of NIL-*SD1* and ZS97 germinated on the same rooting culture medium with a different concentration of GA3 and PBZ for each treatment benches (25 ± 2°C, 55 ± 3% RH, 4800 lux and ten hours photoperiod). Each experiment performed twice per each treatment. The controls were treated with the same conditions free of GA3 and PBZ. Fifteen days later, the length of plantlets height measured from the bottom of the stem to the leaf tip.

## Results

### Characterization of RI92

At the seedling stage of 35 days old, plant height recorded 56 and 40 cm on RI92 and ZS97, respectively (Table 1). RI92 showed a much higher plant height (161 cm) than its parents; ZS97 (86 cm) and MH63 (110 cm) at the maturation stage.

**Table 1:** Plant height (cm) of RI92 and its NIL and parents at seedling and maturation stages.

Stages	RI92	NIL	Zhenshan 97	Minghui 63
Seedling	56.2±5.1	52.7±4.8	40.4±3.0	46±3.5
Maturation	161.0±5.2	140.1±6.6	86.1±4.3	110.2±4.7

10 plants were investigated each genotype.

**Fig 2:**
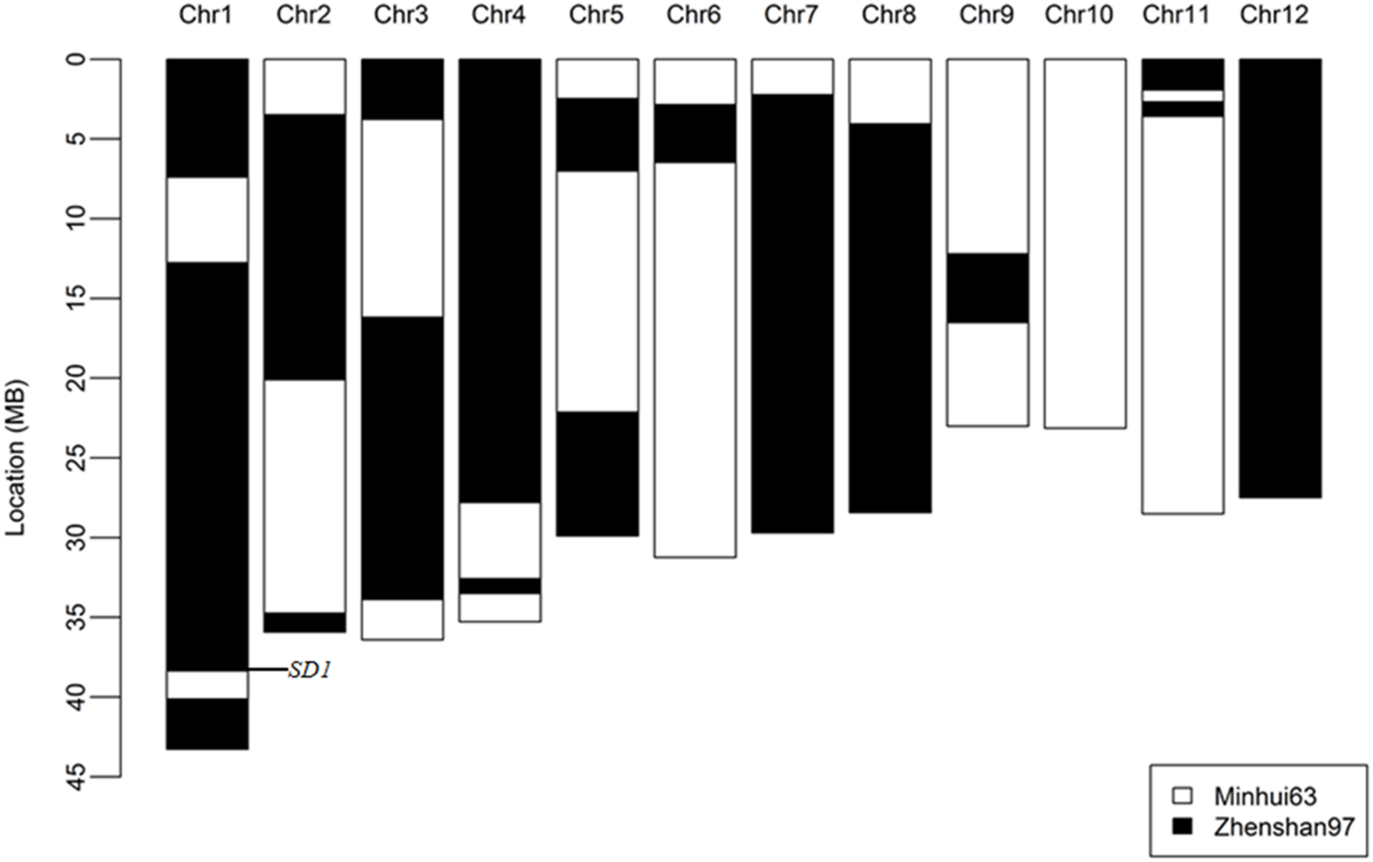
The genetic constitute of RI92.

Because of its extraordinary plant height in the RIL population, it is possible that RI92 contaminated by alien pollen from uncertain genotypes. We proved that the genetic constitution is the mixture of both parents ZS97 and MH63, and no alien genotypes using 221 RFLP and SSR markers [27], meanwhile, we compared its genome constitution with both parents by low-genome coverage re-sequencing [28]. All genome sequences came from both parents without exception (Fig 2). The RI92 genetic background constituted with 34 bins; 55% and 45% of genomic regions were coming from ZS97 and MH63, respectively. At least, one recombination event of each chromosome occurred in RI92 except chromosomes 10 and 12 (Fig 2) which entire originate from ZS97 and MH63 respectively. These data confirmed that RI92 is the offspring of ZS97 and MH63.

### Development of the tall near isogenic lines (NIL)

The plant height of F_1_ derived from the crossing between RI92 and ZS97 was similar to RI92, indicating a dominant gene controlling the tall plant height trait. A BC_3_F_2_ population consisted of 96 plants; the tall NIL in this population was about 10 and 60 cm higher than the recurrent parent (ZS97) at the seedling and mature stage, respectively (Table 1). The NIL had six internodes; two more than ZS97 (each internode of NIL was longer than ZS97). Also the panicle length of NIL was longer than ZS97 panicle length (Table 2).

**Table 2:** Internode length of NIL, ZS97 and RI92.

Terms	RI92	NIL	Zhenshan 97
Panicle length	26.2±3.1	24.0±2.8	22.4±2.3
1^st^ internode	45.8±5.6	40.2±5.2	33.2±2.2
2^nd^ internode	30.5±5.2	27.3±4.7	21.6±1.0
3^rd^ internode	24.9±3.4	21.1±4.5	7.0±1.8
4^th^ internode	18.4±4.3	14.0±6.1	2.6±0.5
5^th^ internode	10.1±4.8	8.4±5.0	-
6^th^ internode	4.5±2.2	4.2±1.8	-

10 plants were investigated each genotype.

**Fig 3:**
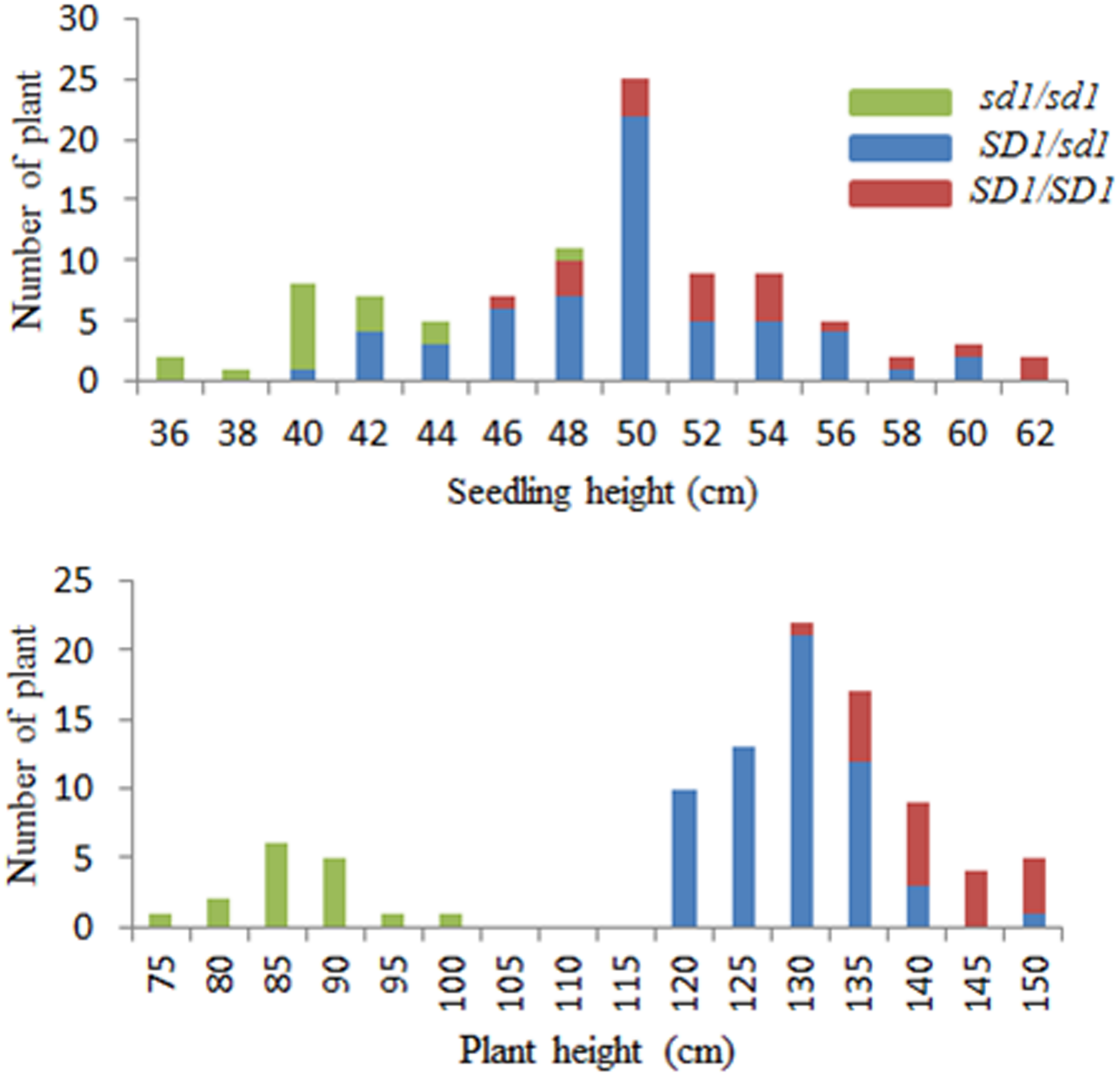
Frequency distribution of seedling height (A) and plant height (B) in the random BC_3_F_2_ population. The red, blue and green bars mean the homozygotes of *SD1/SD1* and *sd1/sd1* types and heterozygote identified by the functional SNP (FNP) in the third exon of *SD1*.

At the seedling stage, the segregation of seedling height in the BC_3_F_2_ population exhibited a continuous distribution (Fig 3A), while the plant height in the mature stage showed a discontinuous distribution with a gap from 100–120 cm (Fig 3B). Obviously, these plants easily classified into two groups: short and tall. The segregation of 16 short plants to 80 tall plants was fit to the segregation ratio of the single Mendelian gene (χ^2^ = 3.56< 3.84, *P* = 0.05), which indicated that a major gene controlling the variation of plant height. Only 2–3 days’ variation of heading date was observed in the BC_3_F_2_ population, suggesting the plant height gene did not impact on heading date.

### Quick mapping for the plant height QTL

**Fig 4:**
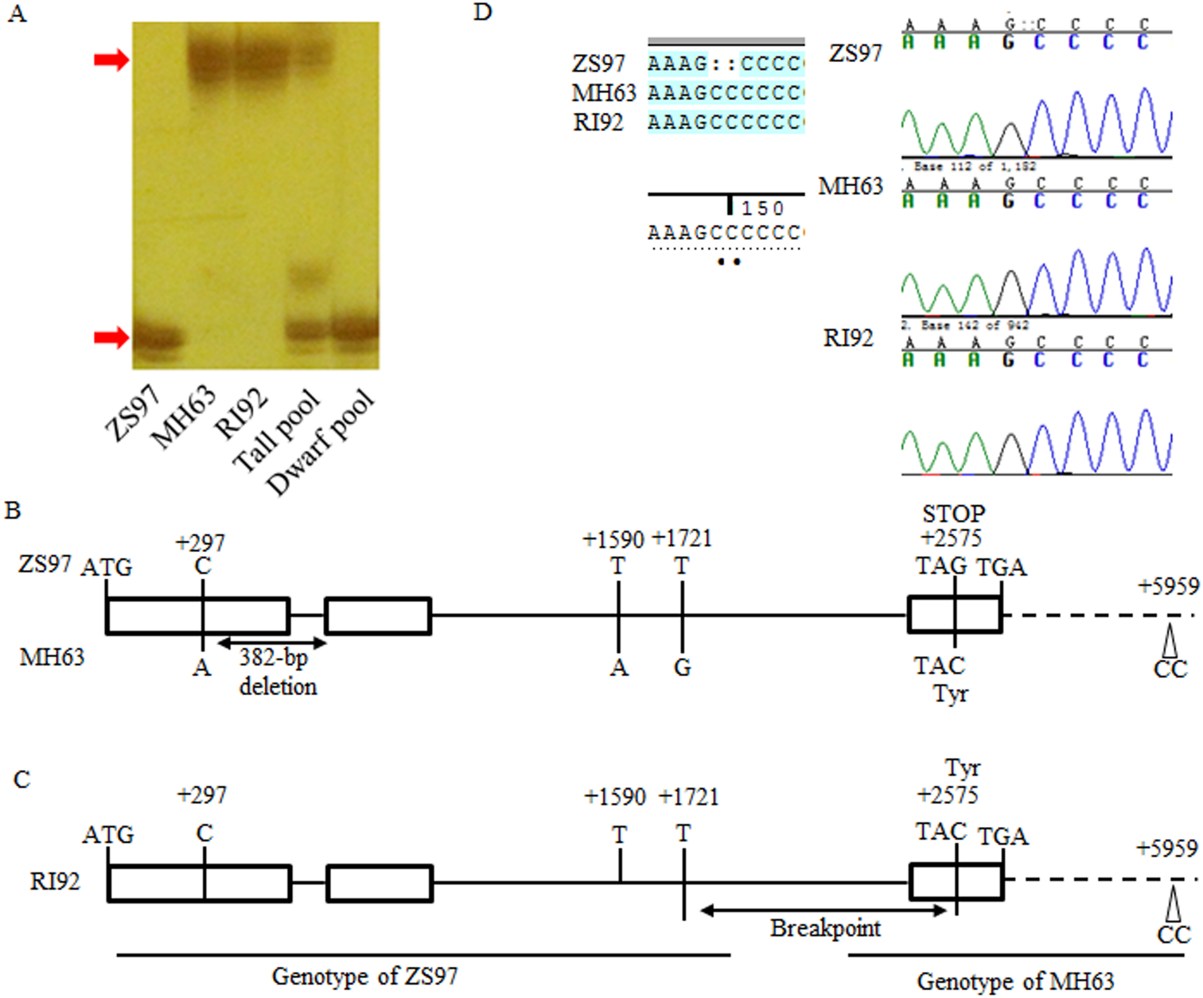
Comparison of the allele sequences of *SD1* among ZS97, MH63 and RI92. (A) Bulk segregant analysis of 10 tall and dwarf plants in BC_3_F_2_ population. The red arrows indicate the target band for ZS97 and MH63 genotypes, respectively (B) Comparison of *SD1* sequences between ZS97 and MH63 alleles. (C) The intragenic recombination of *sd1*, (D) and the same 2-bp insertion in MH63 and RI92.

The difference in plant height is of 60 cm between the tall NIL and ZS97 indicated that the unknown gene had a large effect. In the previous study, many QTLs for plant height mapped in the RILs and Chromosome Segment Substitution Lines (CSSLs) between ZS97 and MH63 [24, 29]. But only one QTL *qPH7/qPH7*.*2* expressed a large effect on plant height in the RILs and CSSLs. Thus, we exclusively focused on the major QTL *qPH7/qPH7*.*2* in the short arm of chromosome 7. But the chromosome region contained *qPH7/qPH7*.*2* was fixed with homozygous ZS97 in RI92. So, the variation of plant height in the BC_3_F_2_ population is not the result of *qPH7/qPH7*.*2*. Then bulk segregant analysis used to quickly identify the major gene. We constructed two DNA bulks for tall and short plants, each bulk consisting of ten plants. *SD1*, a gibberellin synthetase gene in rice, has tremendous effects on plant height. *SD1* was regarded as the possible target gene considering its high effect on plant height in rice. Then the polymorphic SSR markers RM3523 linked to *SD1* were used to genotype the two bulks. PCR results showed two bands and one band for tall and short bulk, respectively (Fig 4A), which suggested that the major gene linked to RM3523, and *SD1* as the most likely candidate.

### Comparative sequencing of *SD1* between parents

Therefore, we sequenced *SD1* for both two parents and RI92. Sequence analysis revealed that there were four SNPs and a 382-bp deletion in the *SD1* gene body between ZS97 and MH63 allele. Two of these four SNPs located in the exon and the other placed in the intron (Fig 4B). The first SNP (+297) which found in the first exon did not lead to any amino acid change. While the second SNP (+2575) of ZS97 in the exon 3 produced a premature termination code, indicating the parent ZS97 possesses non-function *SD1*, that corresponding to the semi-dwarf plant height of ZS97.

Comparing to ZS97, MH63 allele had a 382-bp deletion and the large deletion including the entire first intron and part of the first and second exon. This data indicated that the MH63 allele possessed a non-function *SD1* allele. Therefore, *SD1* was non-functional in both parents. However, unlike both parents, RI92 contained a strong functional *SD1* allele (*SD1-GR*) same as *indica* Kasalath, which had 2 amino acids, differ from Nipponbare or Taichung65 (*SD1-EQ*) (S1 Fig) [21, 23, 30]. The only difference between RI92 and ZS97 allele was the SNP (+2575) guanine (G) substituted to Cytosine (C) (Fig 4C), but the RI92 allele can produce complete gibberellin 20-oxidase protein. The unique SNP at site +2575 between RI92 and ZS97 puzzled us whether the point mutation occurred in the ZS97 allele of *SD1* or a recombination within *SD1* happened between two parents in the process of developing RILs, and resulted in the functional *SD1* allele in RI92. To answer the assumption, 4-kb fragment downstream of *SD1* were sequenced and distinguished between MH63 and ZS97 by a 2-bp insertion (Fig 4C and 4D), while RI92 allele carried the same 2-bp insertion as MH63 contained. Also, the sequence of 2-kb promoter region showed no differences between ZS97 and MH63 alleles.

**Fig 5:**
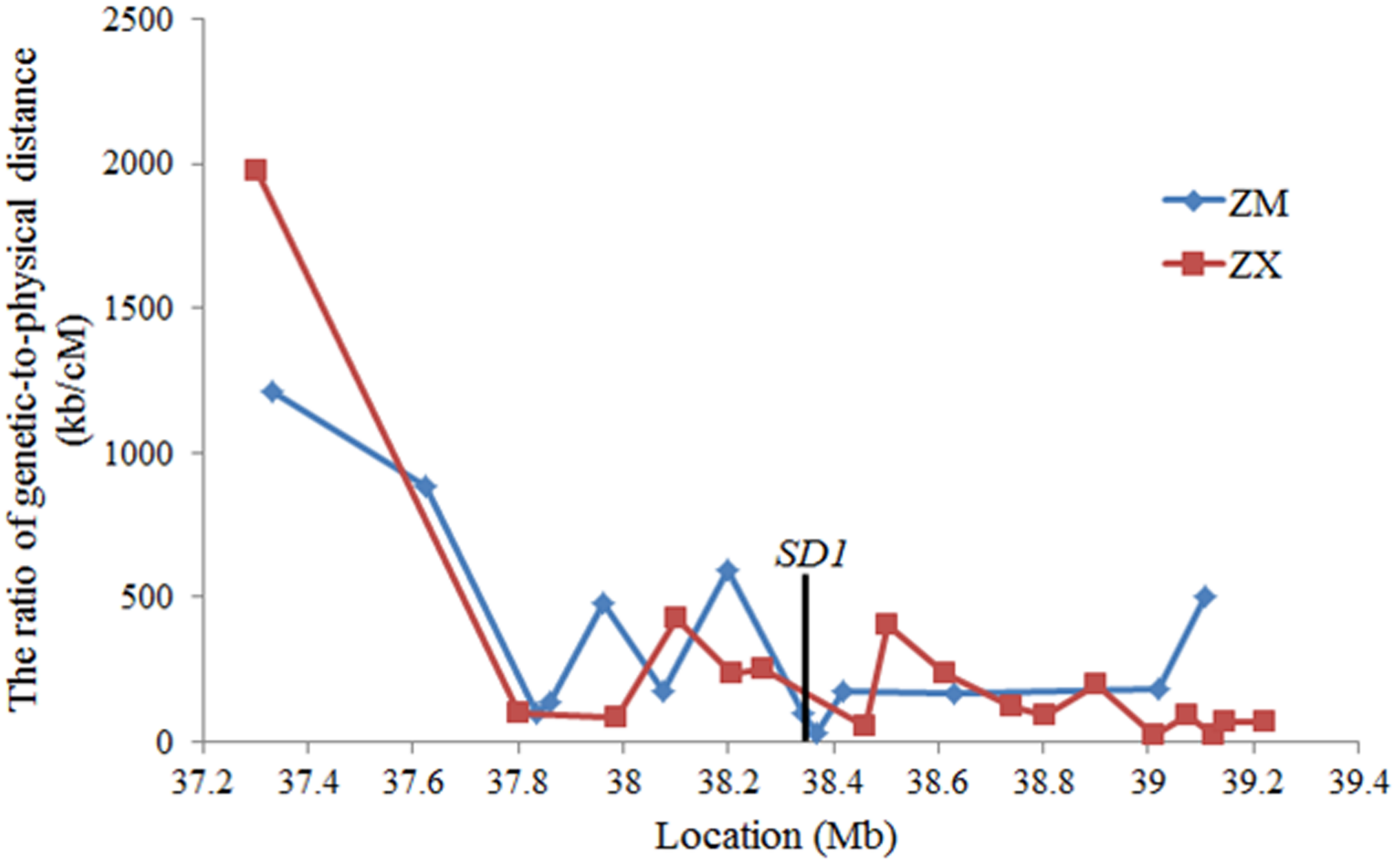
The ratio of genetic-to-physical distance in the 2-Mb region surrounding *SD1* in the RILs of ZM (derived from the cross between Zhenshan 97 and Minghui 63) and ZX (derived from the cross between Zhenshan 97 and Xizang 2).

Recombination rate in the 2-Mb chromosome region surrounding *SD1* calculated based on the bins in the RIL population of 210 lines between two indica cultivars ZS97 and MH63 [11]. The average recombination ratio of physical-to-genetical distance in this region was 220 kb/cM (Fig 5), which was similar to the genome-wide averaged recombination ratio of 250~300 kb/cM [11, 31, 32]. But the ratio for *sd1* located bin was 64 kb/cM. We accordingly estimated the recombination rate in the same 2-Mbregion using a RIL population of 197 lines between indica cultivar ZS97 and japonica cultivar Xizang 2 [12]. The averaged recombination ratio in the whole 2-Mb region was 132kb/cM, while the ratio of *SD1* located bin was 55 kb/cM, this is very similar to that in the indica RIL populations.

### Genetic effects of *SD1* on yield related traits

Now, the possible candidate gene for plant height is *SD1*, and we sequenced it for the 96 BC_3_F_2_ plants. The only SNP G-C at +2575 was the functional nucleotide polymorphism (FNP) of *SD1* [22]. According to the genotype of the FNP, the BC_3_F_2_ population in the maturation stage divided into three classes of CC (*SD1/SD1*), CG (*SD1/sd1*) and GG (*sd1/sd1*). The homozygote *sd1/sd1* plants showed the similar plant height of ZS97 (on average plants 85 cm), whereas the average of homozygote *SD1/SD1* plants and heterozygote *SD1/sd1* plants scored the similar plant height, approximately 140 cm, about 55 cm higher than *sd1/sd1* genotypes (Table 3). Significant differences in plant height between *SD1/SD1* and *sd1/sd1* plants were observed in seedling and maturing stages with *P* values of 1.7E-25 and 2.2E-10 by ANOVA, respectively. We also measured the yield per plant (YP), spikelets per panicle (SPP), grains per panicle (GPP), 1000-grain weight (KGW), panicle length (PL) and biomass per plant. The genotype *SD1/SD1* always had higher significant phenotypic values than the genotypes *sd1/sd1* in all traits except the harvest index (Table 3). Subsequently, the tall plant produces more biomass and grain yield.

**Table 3:** The genetic effects of *sd1* on grain yield related traits in the BC_3_F_2_ population.

Traits	*sd1/sd1*	*SD1/sd1*	*SD1/SD1*	*P* value	*Additive*	*Dominance*	*D/A*
SH (cm)	40.4±3.0	49.3±4.3	52.7±4.8	2.2E-10	6.1	2.7	0.45
PH (cm)	85±5.8	127.5±6.0	139±5.4	1.7E-25	27.0	15.5	0.57
SPP	102±17.7	113±14.2	126±20.6	0.0008	12.0	1.1	0.09
GPP	75±13.9	94±12.7	102±18.6	3.1E-05	13.4	5.3	0.39
PL (cm)	20.2±0.8	22.2±1.0	23.9±1.2	7.5E-12	1.8	0.1	0.08
BM (g)	35.6±11.5	50.5±19.0	57.4±13.6	1.2E-05	10.9	4.0	0.36
YP (g)	17.4±5.5	23.9±9.2	25.8±5.9	0.0001	4.2	2.2	0.54
HI	0.49±0.06	0.47±0.04	0.45±0.04	0.02	0.02	0.002	0.1

SH seedling height, PH plant height, SPP spikelets per panicle, GPP grains per panicle, PL panicle length, BM biomass per plant, HI harvest index, YP yield per plant. The *P* value indicated the significance of difference between homozygote *sd1/sd1* and *SD1/SD1*.

### Functional SD1 plants show sensitive to PBZ

**Fig 6:**
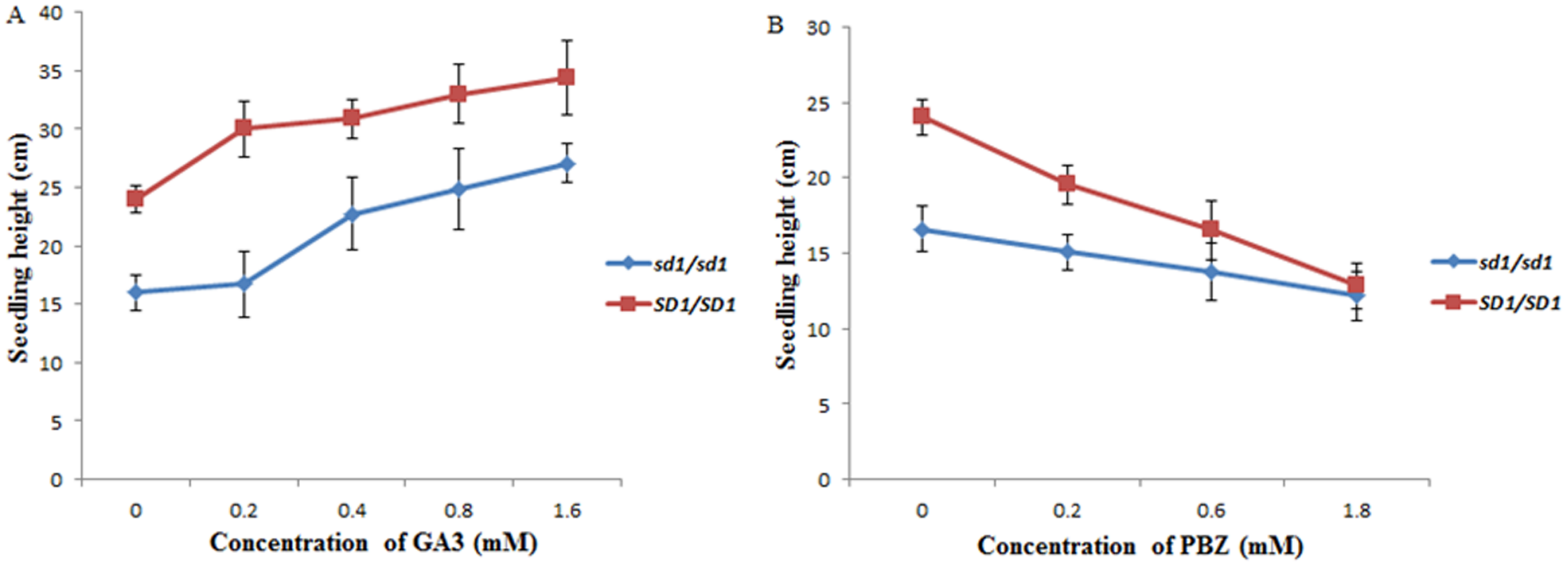
GA3 and PBZ treatment of homozygote *sd1*/*sd1* and *SD1/SD1* plants.

GAs biosynthesis inhibitor, Paclobutrazol (PBZ), was widely used for decreased vegetative growth of rice and increased the chlorophyll content [33]. All treated plants with PBZ showed decreasing seedling heights as compared to the control. With rising of PBZ concentration, the seedling height was further reduced (Fig 6). However, the height reduction rate of *SD1/SD1* plants was higher than those of *sd1/sd1* plants at all concentrations of PBZ while achieving the same reduction at 1.8 mM. These data indicated that the function of *SD1* in *SD1/SD1* plants was completed inhibited by PBZ. On the other hand, the seedling height of *SD1/SD1* plants was rapidly increased after treated with 0~0.2 mM GA3, whereas the seedling height of *sd1/sd1* plants was enhanced sharply with 0.2~0.4 mM GA3.

## Discussion

### Gain-of-function *SD1* allele produced by intragenic recombination led to extraordinary tall plant height in RI92

Plant height as a major factor influencing rice production was well studied in the last two decades. Dozens of QTL for plant height in rice were identified and cloned. The green revolution gene (*sd1*) was widely adopted in semi-dwarf rice breeding for several decades, although the function and mechanization of *sd1* was not clear in that time. Nitrogen fertilization is essential for high yield production, but the unfavourable stem elongation and tall plant height increased the risk of lodging under high levels of nitrogen fertilization. Thus, breeding of semi-dwarf varieties overcome the disadvantage of the nitrogen fertilization, and greatly increased rice production in the world during 1960s-1970s. At least seven types of non-function *sd1* alleles were identified. In this study, the rice cultivar ZS97 possesses the same allele as cultivar 9311 with a premature termination in the third exon [22], and MH63 possesses the same allele as IR8 with a large deletion. Both parents carry non-functional *sd1* alleles. The co-segregation of plant height with the FNP genotype of *SD1* in the BC_3_F_2_ population indicated that *SD1* was the gene underlying the extraordinary tall plant in RI92. GA3 increased the seedling heights of both *SD1/SD1* and *sd1/sd1*; however, the *SD1/SD1* was more sensitive to a lower GA3 concentration. In contrast, PBZ treatment decreased the seedling heights of both *SD1/SD1* and *sd1/sd1*, however, the more decreased heights of *SD1/SD1* with PBZ treatment indicated the higher level of endogenous GAs in *SD1/SD1*. Therefore, endogenous GAs played a critical role in regulating seedling height in *SD1/SD1* plants. According to these results, it is *SD1* that contributes to the tall plant in RI92, although both parents carried non-functional alleles of *sd1*. Hence, gain-of-function of *SD1* occurred in RI92. Most *japonica* cultivars possessed the weak function *SD1-EQ* allele, which resulted either two mutations occurred simultaneously in *SD1-GR* or single mutation happened independently resulting *SD1-ER* and *SD1-GQ* and subsequently occurred intragenic recombination between *SD1-ER* and *SD1-GQ* [23]. In this study, the intragenic recombination between two non-functional *sd1* alleles generated *SD1-GR* allele in RI92, which encoded GA20-oxidase with a higher enzyme activity than the one encoded by *SD1-EQ* allele [23]. That is why RI92 is much taller than both parents.

Sequencing of *SD1* in RI92 showed a functional *SD1* allele, which presented the unique SNP at +2575 in the ZS97 allele. The SNP in ZS97 resulted in a non-functional *sd1* allele by a premature stop codon. Thus, it is a mutant type of non-functional ZS97 allele. On the other hand, ZS97 carried the 5’ end of functional *SD1*, and MH63 carried the 3’ end of functional *SD1*. In theory, the intragenic recombination of *sd1* between parents could produce a functional *SD1* allele. Consequently, either point mutation or intragenic recombination is a possible reason for producing the functional *SD1* allele in RI92. Sequencing of 4-kb fragment downstream of *SD1* identified the same 2-bp insertion in RI92 and MH63 as compared to ZS97. Therefore, the genotypes of RI92 at site +2575 and the 2-bp insertion are the same as MH63. It is almost impossible that both mutations in two sites have simultaneously occurred in one plant. Therefore, we supposed that an intragenic recombination of ZS97 and MH63 *sd1* alleles resulted in a functional *SD1* allele in RI92. Moreover, the recombination breakpoint was located in the region between the site +1721 and the site +2575 because RI92 carried the ZS97 allele at the site +1721 and the MH63 allele at the site +2575 (Fig 4). The 4-fold recombination rate was observed in the *SD1* located bins in both intra-subspecific and inter-subspecific populations. The high recombination rate implemented the occurrence of *SD1* intragenic recombination.

### *SD1* deceased harvest index and increased yield per plant

Since 1960s, the *sd1* was widely used in breeding semi-dwarf varieties, because decreasing plant height can increase lodging-resistant ability and planting number in a given area [34, 35]. In the previous report, null *sd1* with reduced plant height and biomass can increase harvest index. In our study, the *SD1*/*SD1* increased panicle length by 3.7 cm in the BC_3_F_2_ population, spikelets per panicle and grains per panicle are also increased nearly 25 grains comparing to the *sd1/sd1*. Hence, the yield per plant was increased by 8.4 g in the *SD1/SD1* plants. The *SD1/SD1* plants have larger yield per plant and biomass. However, the harvest index is lower than *sd1/sd1* plants. Therefore, functional *SD1* derived from RI92 not only increases plant height but also yield per plant. Of course, such a tall plant faces to a high risk of suffering lodging.

### High mapping resolution would be realized by inter-crossing F_2_ plants

There were 34 bins in RI92, nearly three bins each chromosome. Moreover, chromosomes 10 and 12 were entirely from MH63 and ZS97, respectively. This low number of recombination bins in each plant greatly limited the resolution of QTL mapping, and of course, enlargement of the population size is a way to enhance the mapping resolution. But, this method costs more labors and money. The alternative is increasing the number of bins in each plant, which enhances the mapping resolution in a given population size. The randomly crossing between F_2_ plants and sequentially intercrossing of each line could increase the recombination rate and result in a RIL construction with an improved resolution [36]. Also, the multi-parent advanced generation inter-crossed population may resolve this low recombination rate in a bi-parental RIL population [37].

## Conclusions

We isolated a functional *SD1* allele in a tall recombinant line, RI92, from the offsprings of two semi-dwarf indica cultivars. Sequencing of *SD1* and its flanking region showed an intragenic recombination occurred and the recombination breakpoint located in the region between the site +1721 and the site +2575 of *SD1*. Genome re-sequencing identified only 34 bins in RI92, which limited the QTL mapping resolution in recombinant inbred lines. These results indicated that intragenic recombination could create new alleles in the progeny distinct from parental alleles and diversify natural variation, which provided more chance to select target traits; and increasing population size or changing the crossing style (intercross) would be alternatives to enhancing mapping resolution.

## Supporting information

S1 Fig Comparison of SD1 amino acid sequences among Nipponbare, Taichung65, Kasalath, RI92, ZS97, and MH63. red triangles indicate the two amino acid difference between SD1-EQ and SD1-GR.
(TIF)

S1 Table The primers used for sequencing *SD1*. (DOCX)
