# Supplementary figures and images for: Intragenic recombination between two non-functional semi-dwarf 1 alleles produced a functional SD1 allele in a tall recombinant inbred line in rice
Source: PLoS One. 2017 Dec 27;12(12):e0190116. doi: 10.1371/journal.pone.0190116 (PMC5744974; doi:10.1371/journal.pone.0190116)

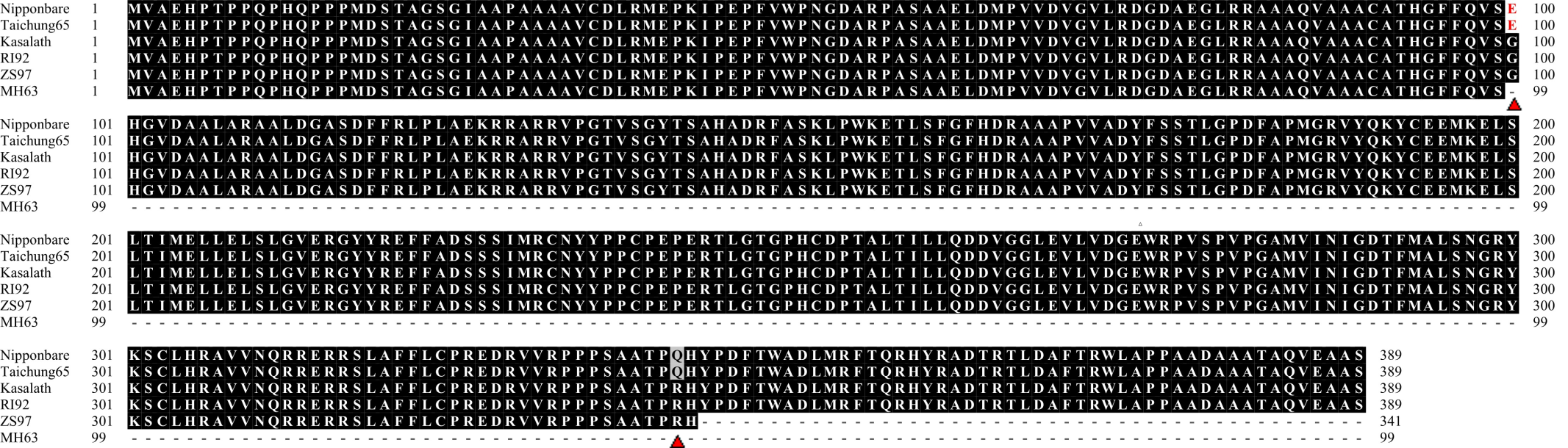

Supplement: S1 Fig — red triangles indicate the two amino acid difference between SD1-EQ and SD1-GR. (TIF) [file pone.0190116.s001.tif]
