# Supplementary material for: Intragenic recombination between two non-functional semi-dwarf 1 alleles produced a functional SD1 allele in a tall recombinant inbred line in rice
Source: PLoS One. 2017 Dec 27;12(12):e0190116. doi: 10.1371/journal.pone.0190116 (PMC5744974; doi:10.1371/journal.pone.0190116)
Supplement: S1 Table — (DOCX) [file pone.0190116.s002.docx]

Supplement Table 1 the primers used for sequencing *SD1*

| Primers | Sequence 5’ to 3’ | Region of *SD1* |
| --- | --- | --- |
| SD1-F | TCTCCCCTGTTACAAATACC | Gene body |
| SD1-1-R | GTAGTTGCACCGCATGATTG | Gene body |
| SD1-1-F | GCGCCAATGGGGTAATTAAAACG | Gene body |
| SD1-R | ACGCGACGAGACACTACTCA | Gene body |
| SD2-F | ATATTTTCCGATGGTGTGACG | Gene body |
| SD2-2-R | TTCCCCACAAATTCCTTCAG | Gene body |
| SD2-2-F | GATTGTTCCATGCCGATCGA | Gene body |
| SD2-R | AGCGCTATCGAACAAAGCAG | Gene body |
| SD1-P1F | TGCATGCAGCCATGTATCTT | 2-kb promoter |
| SD1-P1R | CTTGGGCTCGCAGTAGTTTC | 2-kb promoter |
| SD1-P2F | GGTGGTATAGTGCACCGTGA | 2-kb promoter |
| SD1-P2R | GTGGTTGGAGTGCATTTCCT | 2-kb promoter |
| sd1a-f | GAAACGGAACGAACAGAAGC | 3’ downstream region |
| sd1a-r | CTCCTACGGACGTTGAATCC | 3’ downstream region |
| sd1b-f | GGATTCAACGTCCGTAGGAG | 3’ downstream region |
| sd1b-r | GAGCGTGGCTTGTTTGGTAT | 3’ downstream region |
| sd1c-f | AGCGACTTTACCATGCCTTG | 3’ downstream region |
| sd1c-r | TGGAAAACCAGGAAACGAAG | 3’ downstream region |
| sd1d-f | ACGACTCGTTTCCAATGAGC | 3’ downstream region |
| sd1d-r | TACGCCCTGGTGGTTACTTC | 3’ downstream region |
